# A Multi-Method Approach for the Identification of Social Functioning Profiles in Autistic Adolescents and Young Adults Without Intellectual Disability

**DOI:** 10.1007/s10803-024-06607-9

**Published:** 2024-10-22

**Authors:** Julie Husmann, Clémence Feller, Laura Ilen, Maude Schneider

**Affiliations:** Clinical Psychology Unit for Intellectual and Developmental Disabilities, Faculty of Psychology and Educational Sciences, University of Geneva, Geneva, Switzerland

**Keywords:** Social functioning, Multi-method approach, Neurodevelopmental disorder, Autism spectrum disorder, Latent profile analysis, Heterogeneity

## Abstract

Given the diverse nature of the autism spectrum and the complex, context-dependent nature of Social Functioning (SF), this study aims to delineate profiles of SF in young people with autism. Using a multi-method approach, it aims to gain a comprehensive understanding of social difficulties in people with ASD. This study also examines the co-occurrence of mental health issues within these profiles, which can exacerbate social impairments. This understanding is essential for designing interventions and support systems tailored to the specific needs of people with ASD. 49 autistic individuals aged 12 to 30, without intellectual disability were recruited. A combination of measures was used to thoroughly assess SF. Latent profile analysis was employed to identify distinct profiles of social functioning. A control group of 60 non-autistic people served as a reference for these profiles. Mental health difficulties were evaluated through clinical interviews and questionnaires. Two profiles of SF were identified, illustrating two ways of managing the cost of social interactions. Compared to controls, one was more socially withdrawn, spending more time alone, while the other spent a similar amount of time alone but interacted less with others. A different prevalence of mental health problems was observed within these profiles. This study highlighted two SF profiles in young people with ASD, revealing different approaches to managing social interactions. These results show that people with ASD do not have uniform strengths and difficulties of SF, and that mental health problems exert a significant influence on different aspects of SF.

Social interactions and communication are an integral part of our everyday lives. Whether we are interacting at school, at work, with friends or family, our social skills are fundamental to our ability to function properly. The ability to engage in social interactions has an impact on our development, well-being, academic and professional success, and integration into community life (Rabiner et al., 2016). Therefore, deficits in social functioning and social skills can lead to lower social participation and higher isolation, which are associated with broad negative consequences and poor long-term outcomes (White & Roberson-Nay, 2009). Difficulties in social interaction and communication are a hallmark of Autism Spectrum Disorder (ASD), making it a highly suitable population for understanding impairments in social functioning. ASD is a common neurodevelopmental disorder, with a prevalence of approximatively 1–2% worldwide (Zeidan et al., 2022). Furthermore, although social difficulties encountered by individuals with ASD are present at all ages and developmental levels, they are particularly noticeable in adolescence (Foley Nicpon et al., 2010; Pellicano, 2010). During this transition period, many adolescents with ASD notice that their functioning is somewhat “different” from their peers (Burnett et al., 2009; Crone & Dahl, 2012; Steinberg, 2005). This phenomenon is particularly observable in autistic adolescents without intellectual disability, who are more likely to seek out and initiate social interactions with their peers, and even more so with non-autistic peers, than their counterparts with intellectual disability (ID) (Bauminger et al., 2003). During adolescence, the rules of socialization become more complex, and while parents generally supervise and facilitate their children’s interactions during childhood, adolescents progressively learn to interact more independently (Vernon et al., 2018). Peer relationships also become more important, making adolescents more sensitive to peer acceptance and rejection (Blakemore, 2008; Steinberg & Morris, 2001). This is why a better understanding of social functioning difficulties in ASD during the adolescence period is particularly crucial, when their impact is exacerbated.

Yet, while the presence of social difficulties are part of the diagnostic criteria for ASD, they are also extremely heterogeneous (Jones & Klin, 2009). The best-known example of this heterogeneity is the female phenotype of autism, often characterized by more *masking* and more subtle social impairments compared to their male counterparts (Hull et al., 2020). Mental health is another example of a variable influencing the expression of ASD symptomatology, particularly during adolescence which represents a vulnerability period for the development of mental health difficulties in the general population but also in autism. Higher rates of mood (i.e. depression) and anxiety disorders have been consistently reported in ASD compared to the general population (Hedley et al., 2018; Hossain et al., 2020; Schiltz et al., 2021; Uljarević et al., 2020a; Zeidan et al., 2022). Previous studies have shown that psychiatric comorbidities can significantly increase difficulties in adaptive responses and affect daily activities by diminishing quality of life and accentuating problems such as agitation, passivity, social isolation, aggressiveness, irritability or self-harm (Fitzpatrick et al., 2016; Pan et al., 2023). In other words, the presence of mental health difficulties is likely to influence the expression of autistic manifestations, and by extension, the expression of social impairments (Mannion & Leader, 2013). This heterogeneity among individuals sharing the same diagnosis poses a challenge for medical research and practice. Indeed, it complicates the identification of predictors and therapeutic options, while making the assessment of individual prognosis more difficult (Bzdok & Meyer-Lindenberg, 2018).

Accordingly, the heterogeneity of social difficulties in ASD underlines the relevance of delineating different social functioning profiles in this population. Surprisingly very few studies have attempted to identify more homogeneous subgroups within ASD (Cholemkery et al., 2016; Eagle et al., 2010; Scheeren et al., 2013; Uljarević et al., 2020b; Wing & Gould, 1979). Moreover, it is important to note that in several of these studies, participants with various IQ levels were included, which makes it difficult to determine whether identified profiles were driven by differences in cognitive functioning (Cholemkery et al., 2016; Eagle et al., 2010; Scheeren et al., 2013; Uljarević et al., 2020b; Wing & Gould, 1979). In addition, existing studies have not focused solely on social functioning, but also on other manifestations of ASD, such as language development or theory of mind (Cholemkery et al., 2016; Eagle et al., 2010; Scheeren et al., 2013). To the best of our knowledge, only two studies to date used clustering approaches to identify social functioning profiles (Uljarević et al., 2020b; Wing & Gould, 1979). Uljarević et al. (2020b) explored the existence of homogeneous subgroups of individuals aged between 3 and 17 with ASD using the Stanford Social Dimensions Scale (SSDS). This questionnaire consists of 5 subscales (Social Motivation, Social Affiliation, Expressive Social Communication, Social Recognition, and Unusual Approach) and collects parents’ views on their child’s social skills. The authors identified five different profiles with distinct patterns of strengths and weaknesses in the different dimensions. Another study focusing on social functioning was conducted by Wing and Gould (1979). The authors classified children until 18 years old with ASD, all of whom had moderate-to-severe ID, in 3 different types of social interaction (Social aloofness, Passive interaction and Active but odd interaction) using the MRC Children’s Handicaps, Behaviour and Skills (HBS) structured schedule. During the latter, professional workers such as teachers, nurses, or day-care staff and, for children living at home, a parent (usually the mother) were interviewed (Wing & Gould, 1979). It should be noted that in both these studies, the profiles were either very heterogeneous in terms of cognitive ability, or consisted solely of young people with ID. This again raises the question of whether the profiles identified are induced by differences in cognitive functioning, and whether these profiles would be the same with individuals without an intellectual disability. In short, these attempts to identify profiles of social functioning were based on a single measure (SSDS or HBS) and did not take into account the cognitive level of the participants.

In our view, social functioning is a complex concept encompassing several components, such as social skills, social behavior, and social cognition (Beauchamp & Anderson, 2010) that cannot be fully captured through a single measure. For example, it mobilizes the integration of emotional, linguistic and cognitive skills that are developed from infancy to adolescence (Beauchamp & Anderson, 2010). In addition, social functioning not only requires several skills, but is also extremely context dependent (e.g., one may adopt different behaviors when with family, friends, and strangers). Similarly, an interaction can be subjectively experienced in different ways, and it was found that objective measures of social functioning (such as the percentage of time spent alone) are not necessarily linked to subjective social experiences (Achterhof et al., 2022b). Moreover, subjective social experiences appear to be more strongly related with outcome in adolescents (Achterhof et al., 2022b). This highlights the importance of including the subjective point of view of autistic youths about their social interactions, which has not been done in the previous studies by Uljarević et al., 2020b); Wing and Gould (1979), as only parent-reported measures were used. Hence, the use of a multi-method approach that integrates both ecological and multi-informant (i.e. both subjective and objective) information is particularly well-suited to characterize social functioning in its complexity. Considering the above, an ecological measure capturing information about social interactions in the present moment (e.g., in-the-moment assessments) and how there are experienced seems essential. Therefore, Ecological Moment Assessment (EMA), a self-reported structured diary technique that collects real-life measures in the context of everyday life (Myin-Germeys et al., 2009), appears to be a relevant methodology with high ecological validity for investigating social interactions in everyday life in combination with more “classical” approaches such as the use of parent-reported questionnaires or direct observation by an external examiner.

Considering the above, the aim of this study was firstly to better characterize social functioning in autistic adolescent and young adults through a multi-method approach compared to a sample of non-autistic youths. Secondly, this study aimed to identify different profiles of social functioning within a relatively homogeneous sample of autistic youths without intellectual disability. From a clinical point of view, this would enable to better target therapeutic interventions according to the different social functioning profiles. Finally, this research aimed at better understanding the impact of mental health difficulties on social functioning by examining the distribution of mental health symptoms within the different social functioning profiles. As suggested by the study design, the following hypotheses are primarily exploratory. In terms of social functioning, we expected to identify different profiles of social functioning among the sample of autistic youths. Secondly, given that social difficulties become more pronounced in adolescence, that autistic women often manifest a more subtle autistic symptomatology, and that social functioning is also influenced by mental health difficulties, we expected that gender, age, the number of psychiatric comorbidities and the severity of mental health difficulties would be distributed differently within these different profiles.

## Methods

### Sample

Forty-nine (49% female) individuals with ASD (mean age = 17.85, SD = 4.71) were recruited from clinical centers in Switzerland and France, through a network of healthcare professionals and announcements to family associations. To compare data from these forty-nine individuals with ASD without intellectual disability, sixty non-autistic participants (58% female) (mean age = 18.16, SD = 3.78) were recruited through advertisements at the University of Geneva or through an ongoing longitudinal cohort. Written consent was sought from parents for all participants with ASD, as well as for non-autistic individuals under 18. This study was approved by the Commission Cantonale d’Éthique de la Recherche sur l’Être Humain (CCER) in Geneva (CH). Inclusion criteria for all participants were (1) age between 12 and 30 years, and (2) sufficient command of the French language (fluent verbal communication) as well as sufficient reading skills. Participants with an FSIQ below 70 were excluded from the study. All participants in the clinical group had a confirmed clinical diagnosis of ASD based on the DSM-V criteria. They were assessed using the Autism Diagnostic Observation Schedule, second version (ADOS-2;(Lord et al., 2012), and their parents completed the Autism Diagnostic Interview-Revised with the help of a trained professional (ADI-R; (Rutter et al., 2003). For non-autistic participants, exclusion criteria included (1) being born prematurely, (2) having a first-degree relative with a developmental disorder, (3) having a lifetime history of psychiatric, neurological, or learning disabilities. All participants were assessed using the Wechsler intelligence scales for children (WISC-V; (Wechsler, 2014) or WAIS-IV; (Wechsler, 2011). The descriptive characteristics of the two groups are presented in Table 1.

**Table 1:** Sample characteristics

	Diagnostic groups		Social functioning profiles within ASD sample	
	Non-autistic	ASD	Profile 1	Profile 2
*N*	60	49	20	29
Gender (female (%))	35 (58.33%)	24 (48.98%)	11 (55%)	13 (44.83%)
Age (mean (SD))	18.16 (3.7868)	17.85 (4.7164)	19.11 (5.1441)	16.98 (4.1817)
Full Scale IQ (mean (SD))	112.8 (12.1160)	108.67 (13.5003)	109.25 (15.4689)	108.27 (11.9392)
**Mental Health difficulties**				
Externalizing (ABCL/CBCL) (mean (SD))	46.67 (10.4907)	58.45 (9.7939)	50.55 (6.7266)	63.89 (7.6264)
Internalizing (ABCL/CBCL) (mean (SD))	49.5 (9.6099)	71.92 (11.6195)	68.70 (11.7435)	74.14 (10.9975)
Externalizing (ASR/YSR) (mean (SD))	52.55 (9.6547)	55.71 (10.7532)	53.05 (10.5663)	57.55 (10.4933)
Internalizing (ASR/YSR) (mean (SD))	51.20 (9.8806)	69.02 (10.9609)	69.30 (8.9838)	68.83 (12.1345)
**Psychiatric diagnosis (N (%))**				
Mood disorder			4 (20%)	12 (41.38%)
Anxiety disorder			9 (45%)	15 (51.72%)
Obsessive-Compulsive disorder			0 (0%)	2 (6.9%)
Other neurodevelopmental disorder (N (%))			3 (15%)	11 (37.93%)
ADHD			3 (15%)	10 (34.48%)
Tic disorder			0 (0%)	3 (10.35%)
Oppositional defiant disorder			0 (0%)	3 (10.34%)
Mean number of psychiatric comorbidities			1.2	2
**Psychotropic medication** **(N (%))**			5 (25%)	10 (34.48%)
Antidepressants			3 (15%)	6 (20.70%)
Antipsychotics			1 (5%)	3 (10.34%)
Anxiolytics			3 (15%)	1 (3.45%)
Psychostimulants			0 (0%)	2 (6.9%)

*Note* The same participant can have > 1 psychiatric diagnoses, neurodevelopmental disorders and medication

### Procedure and Assessment

The current research was carried out as part of a larger study also involving other tasks that are not described here. All participants received a 90.- Euro voucher for their participation to the study. This study was co-registered on the OSF platform (10.17605/OSF.IO/S8YVP) and the data are available open access on the Yareta preservation system. Deviations from the original statistical analysis plan are noted in supplementary material.

### Social Functioning

Social functioning was assessed using a combination of approaches, including self- and parent-assessment, as well as direct observation by an independent examiner.

### Self-evaluation: Ecological Momentary Assessment

The Ecological Momentary Assessment (EMA) is a structured self-evaluation diary. A questionnaire is completed over six days with eight semi-random notifications per day via a cell phone application. Further information on the EMA protocol can be found in supplementary material. In line with previous studies and general recommendations (Myin-Germeys et al., 2009; Palmier-Claus et al., 2011), only participants who responded to at least a third of the beeps were retained in the analyses. At each beep, the same momentary EMA questionnaire was delivered. It comprised a minimum of thirty-three items and a maximum of thirty-eight items, depending on the answers to the conditional questions. Regarding social functioning, participants were asked to indicate whether they were alone or in the company of others. The percentage of time spent alone over the 6-day assessment period was then calculated for each participant (time spent alone). If participants indicated being alone, they were asked about their appreciation of aloneness, their feelings of isolation and rejection, and their desire to be with other people (appreciation of aloneness). If they reported to be in the company of other people, they were asked about their appreciation of the company of others, their feelings of judgment and nervousness, and their desire to be alone (appreciation of company). They were also asked how engaged they were in interacting with that company (level of interaction). The exact wording of each item used to measure social functioning can be found in supplementary material. There were no open-ended questions, and all questions were composed of a 7-point Likert scale (1 = not at all and 7 = extremely). A Cronbach’ alpha coefficient was performed to ensure that the items making up the different variables (i.e., appreciation of aloneness and appreciation of company) loaded onto a single component (values greater than > 0.60). In this context, feeling of rejection was therefore removed from the variable “appreciation of aloneness”, as the value was 0.51. It should also be noted that items relating to the desire to be with other people (from the variable *appreciation of aloneness*), the desire to be alone and the feelings of judgment and nervousness (from the variable *appreciation of company*) were reversed in the analyses to simplify the interpretation, with lower scores reflecting lower subjective appreciation of aloneness and company.

### Parent-reported Questionnaires

Two parent-reported questionnaires were used to evaluate social functioning. The Social Responsiveness Scale (SRS-II) identifies social impairments associated with ASD and quantifies their severity. Various behaviors observed in natural environments are evaluated using a 4-point Likert-type scale response format, ranging from *not true* to *almost always true*. This questionnaire exists in two versions, the School-Age Form for children and adolescents under 18 years and the Adult-Form for adults aged 18 years or more. Items on each form differ slightly to accommodate the age ranges tested; however, there is considerable overlap across both forms (Bruni, 2014). Both versions are composed of sixty-five items and give a total score reflecting the severity of overall social difficulties, as well as scores on five subscales: *Social awareness*, *Social cognition*, *Social communication*,*Social motivation*, *Restricted interests and repetitive behavior*. In addition, the SRS-II offers two DSM-5 compatible subscale; *Social Communication and Interaction* and *Restricted Interests and Repetitive Behavior*. The SRS-II total score was used in the current study, and it should also be noted that this score was reversed in the analyses to facilitate interpretation, with higher scores representing better social functioning.

The Emotion Regulation and Social Skills Questionnaire (ERSSQ) is a parent-report questionnaire consisting of twenty-seven questions designed to assess their child’s behaviors, which are rated in terms of frequency on a 5-point Likert scale from *never* to *always*. This questionnaire exists in two versions, one for children and adolescents under 18 years and the other for adults aged 18 years or more. Items are summed to provide a total score representing the child’s social skills, with higher scores reflecting higher social skills (Beaumont & Sofronoff, 2008).

### Direct Observation by an External Examiner: Role-Play

We used an adapted version of Social Skills Performance Assessment (SSPA), a social role-play task displaying two short scenarios presenting social problem situations (Patterson et al., 2001; Verhoeven et al., 2013). For each scenario, participants are asked to perform a 3-minute role-play with a trained examiner. The SSPA is composed of a practice scene which is not scored but serves to familiarize the participant to the setting. The first scenario involves meeting a new neighbor (played by the examiner) with the aim to greet him and get to know him in a friendly and informative way. The second scenario involves calling a classmate (played by the examiner) who borrowed a notebook, with the aim to get the notebook back while remaining polite. The role-plays were videotaped for scoring and each scene, except the practice one, was double-scored by two examiners on a series of relevant dimensions using a 5-point Likert scale (for additional information, see supplementary material). The SSPA total score was used in the analyses, with higher scores reflecting more effective social skills.

### Psychiatric Diagnoses and Mental Health

A structured clinical interview using the Kiddie Schedule for Affective Disorders and Schizophrenia – Present and Lifetime version (K-SADS-PL) or the Structured Clinical Interview for Axis I DSM-V (SCID) was conducted with participants with ASD and their parents by a trained psychologist during two separate interviews to identify the presence of co-occurring mental health difficulties. These were grouped into 5 categories: Mood disorder, Anxiety disorder, Obsessive-Compulsive disorder, Other neurodevelopmental disorder (Attention Deficit Hyperactivity Disorder (ADHD) and Tic disorder), and Oppositional defiant disorder. We also used the total number of psychiatric diagnoses as an indicator of psychiatric comorbidity (Sandini et al., 2020)(Psychiatric diagnosis are available in Table 1). A more dimensional measure of mental health difficulties was also used with self-reported and parent-reported questionnaires. The Adult Self-Report (ASR) for adults aged 18 to 59 or the Youth Self-Report (YSR) for children aged 11 to 18 is a self-reported questionnaire containing 126 items assessing eight sub-domains (i.e., Withdrawn, Somatic Complaints, Anxious/Depressed, Social Problems, Thought Problems, Attention Problems, Delinquent Behavior, and Aggressive Behavior). Items are rated on a 3-point scale from *not true* to *very true*. The Adult Behavior Checklist (ABCL) for adults aged 18 to 59 or the Child Behavior Check List (CBCL) for children aged 6 to 18 is a questionnaire completed by parents about their child. This 126-item questionnaire assesses the same dimensions as the ASR/YSR (Achenbach, 2001; Achenbach & Rescorla, 2003). ASD research found that three of the eight sub-domains constitute the higher-order *internalizing* scale (i.e., anxiety/depression, somatic complaints, and withdrawal), and two other sub-domains comprise the *externalizing* scale (i.e., delinquency problems and aggressiveness) (Bölte et al., 1999). Consequently, the scores of internalizing and externalizing problems of the two types of questionnaires (self- and parent-reported) were used in this study. A *t*-score ≥ 65 indicates clinical symptoms.

### Statistical Analyses

Statistical analyses were conducted using R version 4.2.1. For all analyses, the level of statistical significance was set to *p* < 0.05. Group comparisons for the descriptive analysis were explored using a Mann-Whitney *U* test or a chi-square test. A latent profile analysis (LPA) was performed to identify profiles of social functioning. LPA identifies latent profiles based on averages of continuous observed variables (Gana et al., 2022). In psychology, LPA is an increasingly used analysis technique, especially when there is a certain amount of heterogeneity between participants and this variation cannot be explained by known variables (Wolfe, 1970). LPA can then be used to identify participants’ profiles, helping to better identify and understand the heterogeneity of a sample (Sterba, 2013). In this study, a LPA was conducted using social functioning variables to extract social functioning profiles from the ASD population. Firstly, the EMA self-evaluation variables: time spent alone, appreciation of aloneness, appreciation of company, level of interaction. Secondly, the scores of the parent-report variables: SRS-II and ERSSQ and thirdly the score of the examiner-report variable: SSPA. First, as the goal of this study was to identify social functioning profiles with the autism spectrum, only participants with ASD were included in the LPA. LPA analysis was performed for one to seven latent classes, the total number of variables measuring social functioning. The optimal number of profile was selected regarding six model fit indexes, such as the Akaike Information Criterion (Akaike, 1974), the Bayesian Information Criterion (Schwarz, 1978), the Bootstrapped Likelihood Ratio Test (BLRT), and the Entropy index (Nylund et al., 2007; Ramaswamy et al., 1993). AIC and BIC are goodness of fit measures to compare different counterpart models with lower values suggesting better model fit (Nylund et al., 2007). BLRT with a significant p value under or egal 0.05 signals that the k-class model is superior to the k-1 class (Dziak et al., 2014). The entropy index provides a measure of classification accuracy, with a value exceeding 0.80 acceptable and a higher value indicating better accuracy. Robust regressions were used to examine differences in terms of social functioning variables between the profiles, controlling for age, gender, and IQ and between each profile and the control group which is composed of non-autistic individuals. The severity of mental health difficulties was compared across the different social functioning profiles using robust regressions, again controlling for age, gender, and IQ. Logistic and ordinal regression were used to compare the frequency of psychiatric comorbidities across profiles.

## Results

### Sample Characteristics

Participants with ASD did not differ from non-autistic participants in terms of age (*U* = 1268.5, *p* = 0.35), gender (*X*^*2*^(1) = 0.95, *p* = 0.32) and IQ (*U* = 1110, *p* = 0.09). Regarding mental health difficulties, participants with ASD were characterized by more severe internalizing problems using both self- (*U* = 2595.5, *p* < 0.001) and parent-reported evaluation (*U* = 2742.5, *p* < 0.001). For the externalizing problems, participants with ASD had higher scores than non-autistic participants only according to the parent-evaluation (ABCL/CBCL) (*U* = 2302, *p* < 0.001) Note that for individuals with ASD, the scores of internalized problems were in the clinical range (*t*-score ≥ 65) but those of externalized problems were not.

### Identification and Description of the Best-Fitting Latent Profile

The results of model fit indices from the APL (from one-profile to seven-profile solutions) are provided in Table 2. Although the four-profile solution had a lower AIC, the other fit indices were not as good as the two and three-profile solutions. Furthermore, the three-profile solution did not provide better model fit indices than the two-profile solution, except for the AIC. In addition, the AIC is recognized as a criterion that may overestimate the number of underlying profiles (McLachlan & Peel, 2000; Morgan et al., 2016; Tofighi & Enders, 2008). The BIC, on the other hand, is standardized to favor models with high likelihood and smaller sample sizes (Tofighi & Enders, 2008). The two-profile solution gathered most criteria of model fit indices. It had a lower BIC but also a higher entropy than the requested size (> 0.80) and a significant p-value of BLRT (0.01). Therefore, the two-profile solution was selected. The characteristics of the two different profiles of social functioning emerging from this APL are available in Table 1.

**Table 2:** Model fit indices

Classes (Profiles)	AIC	BIC	Entropy	BLRT
1	994.32	1020.80	1.00	
2	960.96	1002.58	0.97	0.01
3	957.66	1014.41	0.91	0.09
4	948.83	1020.72	0.92	0.03
5	954.07	1041.09	0.86	0.60
6	947.44	1049.59	0.89	0.04
7	935.32	1052.62	0.94	0.01

### Between-Group Differences in Terms of Social Functioning

**Fig. 1:**
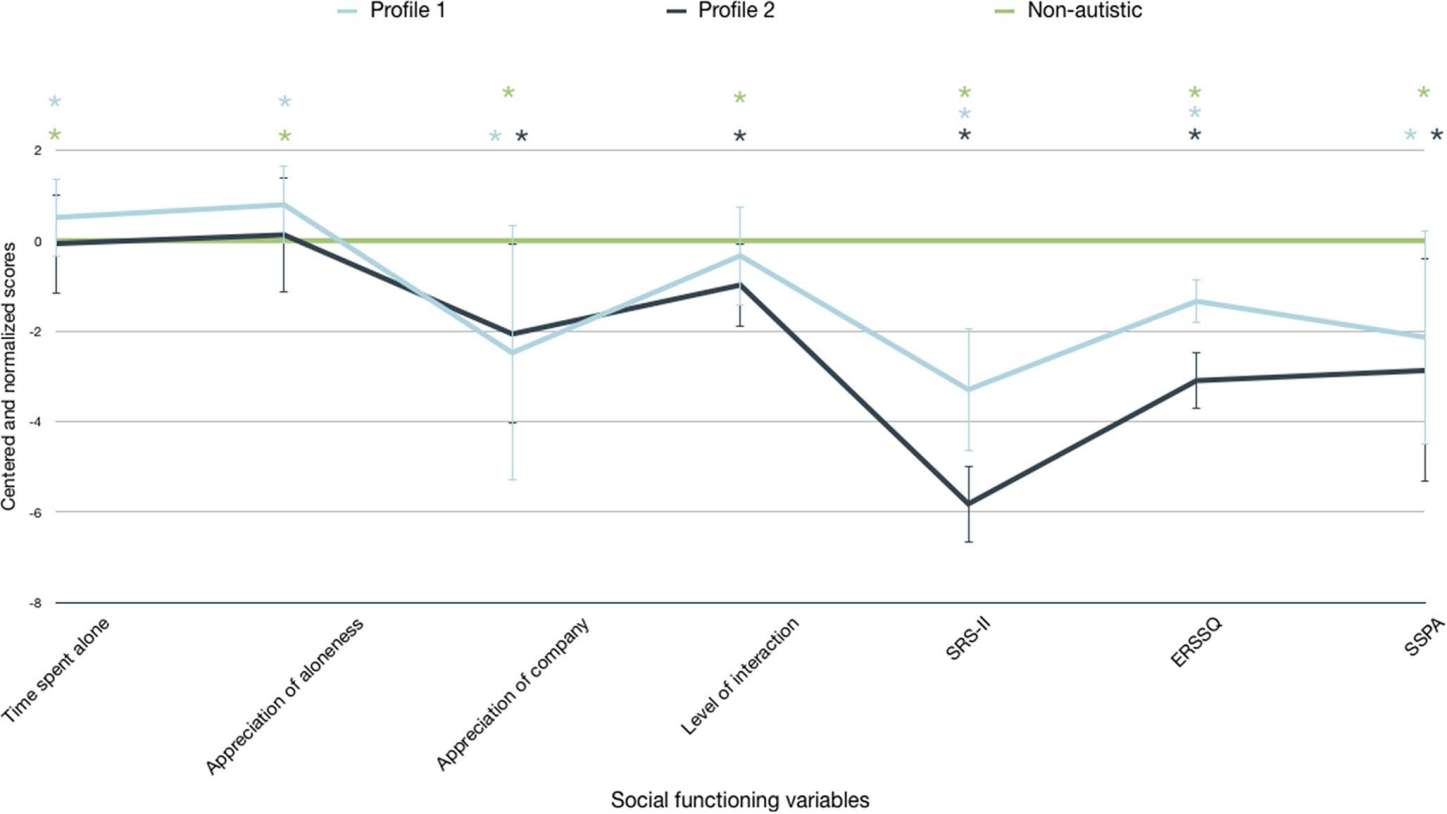
Social functioning profiles for the entire sample using a multi-method approach. *Note*: To facilitate the readability and the comprehension of the graph, the non-autistic participants (control group) were used to calculate standardized z scores and scores of autistic youths are reported as comparisons from this control group. The results of the APL are available in supplementary material

A comparison of the different social functioning measures across the two profiles are presented in Fig. 1. The two profiles did not differ in terms of age (*U* = 354, *p* = 0.12), gender (*X*^*2*^*(1)* = 0.49, *p* = 0.48), and IQ (*U* = 295.5, *p* = 0.75).

Compared to the control group, Profile 1 is characterized on the ESM variables by a greater percentage of time spent alone (*t*(76) = -1.000, *p* = 0.03), a greater appreciation of aloneness (*t*(76) = 3.236, *p* = 0.002) and a lower appreciation of the company of others (*t*(76) = -3.550, *p* < 0.001). According to the variables reported by their parents, notably the total score on the SRS-II and the total score on the ERSSQ, youths from Profile 1 showed reduced reciprocity (*t*(76) = -8.826, *p* < 0.001) and, as well as lower emotional and social regulation skills (*t*(76) = -9.543, *p* < 0.001) than those in the non-autistic group. They were rated by examiners as having lower social skills during role-plays (SSPA total score) (*t*(76) = -3.604, *p* < 0.001). The two groups did not differ on the level of interaction (*t*(76) = -1.042, *p* = 0.30).

Compared to the control group, Profile 2 was characterized on the ESM variables by a lower appreciation of the company (*t*(85) = -4.915, *p* < 0.001) and a lower level of interaction (*t*(85) = -4.181, *p* < 0.001). On the variables assessed by parents, Profile 2 individuals had less social reciprocity (SRS-II) (*t*(85) = -26.438, *p* < 0.001) and fewer emotional regulation and social skills (ERSSQ) than controls (*t*(85) = -20.651, *p* < 0.001). Examiners also assessed lower social skills in Profile 2 individuals compared to controls (*t*(85) = -7.779, *p* < 0.001). The two groups did not differ on time spent alone (*t*(85) = 2.141, *p* = 0.94) and the appreciation aloneness (*t*(49) = 0.939, *p* = 0.35),

The two ASD profiles differed from each other only on parent-reported variables. Parents reported more social reciprocity (SRS-II) (*t*(45) = -6.222, *p* < 0.001) and better emotional regulation and social skills (ERSSQ) (*t*(45) = -9.720, *p* < 0.001) in youths from Profile 1 compared to those from Profile 2. Individuals therefore had the least adaptive social functioning, according to their parents. Non-significant results are available in supplementary material.

### Post-hoc Analyses

Post-hoc analyses were conducted to better understand and characterize the differences across the two profiles. Firstly, as significant differences across the two profiles were only visible in parent-reported measure, notably the SRS-II, a more qualitative analysis of these differences using the five subscales comprising the SRS-II was conducted. A robust linear regression first revealed significant differences between the two profiles on the following dimensions of the SRS-II, with individuals from Profile 1 having consistently less difficulties than those from Profile 2: Social awareness (*t*(45) = -4.247, *p* < 0.001), Social cognition (*t*(45) = -4.981, *p* < 0.001), Social Communication (*t*(45) = -6.558, *p* < 0.001), and Restricted interests and repetitive behavior (*t*(45) = -4.636, *p* < 0.001). However, no significant difference was observed on the dimension of Social motivation (*t*(45) = -1.370, *p* = 0.18).

Secondly, the two profiles of social functioning could be the result of different socialization contexts. Based on the results of the EMA assessment, the percentage of time spent in various compagnies was computed for each participant: (1) people they are living with (including pets); (2) familiar persons they don’t live with (family members, boyfriend/girlfriend, friends, colleagues/classmates); (3) unfamiliar persons (health professionals, acquaintances, strangers). Details of these categories are available in supplementary material. The results show no significant differences between the two profiles in the type of company, whether it is people they live with (1) (*t*(45) = 1.639, *p* = 0.11), familiar persons (2) (*t*(45) = 1.392, *p* = 0.17), or unfamiliar persons (3) (*t*(45) = -0.088, *p* = 0.93). However, it is worth noting that individuals in the control group (non-autistic) spend less time with people they live with (1) compared to both Profile 1 (*t*(76) = 2.764, *p* = 0.007) and Profile 2 (*t*(85) = 2.544, *p* = 0.01). On the other hand, non-autistic individuals spend more time with familiar persons (2) than individuals in Profile 1 (*t*(76) = -2.892, *p* = 0.005) and Profile 2 (*t*(85) = -2.733, *p* = 0.007). No difference was observed regarding the time spent with unfamiliar persons (3) between non-autistic individuals and Profile 1 (*t*(76) = -0.437, *p* = 0.66) as well as between non-autistic individuals and Profile 2 (*t*(85) = -0.453, *p* = 0.65).

### Association Between Latent Profiles and Mental Health

An ordinal logistic regression analysis revealed that individuals with ASD who have more psychiatric comorbidities are more likely to belong to Profile 2, which is associated with a less adaptative social functioning (OR = 3.6506; 95% CI = 1.793 to 7.482; *p* = 0.02). This analysis also indicated that the types of psychiatric comorbidities do not predict membership in different profiles. These results are available in supplementary material. In addition, a robust regression analysis comparing the severity of mental health difficulties between the two profiles revealed more severe externalizing problems, as reported by parents (ABCL/CBCL), in individuals from Profile 2 compared to Profile 1 (*t*(45) = 6.163, *p* < 0.001). Note that a trend was observed for parent-reported internalizing problems (*t*(45) = 1.873, *p* = 0.06). No significant differences were observed for self-reported (ASR/YSR) internalizing problems (*t*(45) = 0.908, *p* = 0.37). However, a trend was observed for self-reported externalizing problems (*t*(45) = 1.866, *p* = 0.07). Finally, no difference in terms of psychotropic medication was observed between the two profiles. These results are available in supplementary material.

## Discussion

The aim of this study was to better characterize the social functioning profiles of young people with autism without intellectual disability. A multi-method assessment enabled us to discern different dimensions of social functioning, both objective and subjective, which contribute to the distinction of these profiles. Our main results indicate the existence of two profiles of social functioning. These two profiles are similar in some aspects and differ from each other mainly on measures reported by parents. Moreover, both profiles are also similar to non-autistic individuals on certain aspects of social functioning. For example, individuals from Profile 2 spend as much time alone and appreciate solitude as much as the non-autistic group. Profile 1, on the meantime, has the same level of interaction as the non-autistic group. Finally, belonging to one of these two profiles was strongly associated to the presence or absence of mental health difficulties.

Compared with the non-autistic group, both ASD profiles appreciated the company of others to a lesser extent (EMA) and were rated by examiners as less socially skilled (SSPA). Both groups also showed lower social skills than the control group according to parents (SRS-II and ERSSQ). More specifically for each profile, individuals from Profile 1 were more withdrawn, spent more time alone and had a greater appreciation of aloneness than non-autistic individuals (EMA). On the other hand, individuals from Profile 2 had a lower level of interaction than the control group (EMA). Finally, the two ASD profiles differed from each other in terms of the severity of social impairments based on the parent-report (SRS-II and ERSSQ). In fact, the parents of individuals in Profile 2 rated their social skills as less good than the parents of individuals with Profile 1. Individuals with ASD thus manifest their social difficulties in different ways: some individuals are more socially withdrawn, spend more time alone and appreciate being alone (Profile 1), while others don’t spend more time alone, but have a lower level of interaction and tend to have a lower appreciation of other people’s company (Profile 2).

While some studies have considered social withdrawal to be an inherent feature of ASD (Bauminger et al., 2003; Jawaid et al., 2012; Wallace et al., 2017), others have shown the opposite, pointing out that people with ASD do not necessarily spend more time alone than their non-autistic peers (Feller et al., 2022; Hintzen et al., 2010). The existence of two profiles in the present study therefore brings important information regarding this discrepancy. Comparing these two profiles with those identified in Wing and Gould’s (1979) article, we find a certain similarity with two of the four types of social interaction quality in their study. The *Passive* interaction type describes individuals who do not spontaneously establish social contact but accept approaches from others in a friendly manner and adopt a rather adaptive social response. This type of interaction could correspond to Profile 1, in which individuals are rather socially withdrawn but interact and appreciate the company of others to the same extent than their non-autistic peers, adopting slightly lower social skills than their non-autistic peers that allows them to interact relatively fluently with others. The *Active but Odd* interaction type is characterized by individuals who establish interactions spontaneously, but whose interaction is less adapted because it is more self-focused. These children were sometimes rejected by their peers because of this behavior (Wing & Gould, 1979). This type of interaction could correspond to Profile 2, where individuals report more opportunities to be socially active, but also find it more difficult to connect with their peers. Indeed, according to the same study, this type of interaction groups together individuals whose behavior is rather egocentric and uninterested in the needs and ideas of others (Wing & Gould, 1979). Note that this is consistent with the lower level of interaction reported by individuals from Profile 2 compared with the control group. Individuals from Profile 2 could therefore be considered to be socially active, but with pronounced social difficulties in terms of reciprocity (Level of interaction, SRS-II). These two profiles clearly illustrate the different ways in which individuals with ASD manage their social relationships, which entail a considerable cognitive cost (Barendse et al., 2018; Bauminger et al., 2003; Crone & Dahl, 2012). Some spend more time alone, while others tend to withdraw from social interactions. It should be added that both profiles have similar IQ levels, suggesting that the different expression of social difficulties does not depend on cognitive ability. This is consistent with previous studies of people with ASD, which have reported no significant association between IQ and adaptive functioning (Klin et al., 2007; Perry et al., 2009). It is also important to note that the *Aloof* type originally described by Wing and Gould (1979) was not observed in our sample. This relational profile seems to be mainly observed in autistic individuals with intellectual disability, which does not correspond to the characteristics of our group. In addition, it is possible that some individuals, who might initially have been classified as *Aloof*, have evolved towards a more *Passive* type due to their ability to mask their social difficulties. This hypothesis relates to the concept of masking that is largely discussed in the context of ASD and that has been more frequently reported in adolescents than children (Hull et al., 2017; Lai et al., 2017; Alice Ross, Rachel Grove, & John McAloon, 2023). Compared to the study by Wing and Gould (1979), which included children from 2 years to 18 years, our sample was composed of older and less heterogeneous individuals. The characteristics of our sample may also explain the absence of significant age differences that was expected, given that social difficulties become more pronounced in adolescence (Bauminger et al., 2003; Foley Nicpon et al., 2010). Furthermore, and contrary to our hypotheses, no gender differences were observed between the profiles. This is surprising, given that autistic women often manifest a more subtle autistic symptomatology. Overall, and despite a homogeneous sample in terms of socio-demographic characteristics, the existence of these two profiles highlights the heterogeneity of the autism spectrum and the importance of a using a multi-method evaluation to characterize social functioning.

Curiously, individuals from the two profiles differed from each other only regarding parent-reported measures of social functioning. One could think that this result is driven by different socialization contexts (e.g. different amount of time spent alone or with familiar persons). However, post-hoc analyses revealed no significant difference in terms of social contexts between the two profiles. In line with a previous study with an overlapping sample (Feller et al., 2022), non-autistic individuals spent more time with familiar people (e.g., friends) and less time with people they live with, in contrast to autistic participants who spent more time with people they live with than with familiar people. Indeed, in the current study, this was observed for autistic individuals in both profiles. In order to better understand the qualitative differences across the two profiles, a post-hoc analysis was conducted on the different dimensions of the SRS-II. The results showed significant differences for all dimensions, with individuals from Profile 1 having consistently fewer difficulties than those from Profile 2, except for the social motivation dimension for which the two groups had similar scores. These results suggest that individuals from Profile 1 have overall better social skills according to their parents compared to those from Profile 2, but a similar level of motivation to interact socially. Although the two profiles interact in different ways, they are both equally motivated to engage in social interactions. Moreover, preserved motivation for interpersonal interactions in youths with ASD has been highlighted in previous studies (Bauminger et al., 2003; Deckers et al., 2014; Feller et al., 2022). This reinforces the importance of integrating different measurement methods when assessing complex concepts such as social functioning.

Interestingly, the presence of mental health difficulties strongly discriminated individuals from the two profiles, with youths from Profile 2 consistently reporting more severe mental health difficulties. This is consistent with the fact that the presence of psychopathology significantly modulates the social behavior of people with ASD (Mannion & Leader, 2013). It is likely that the association between mental health and social functioning is bidirectional, as suggested by some previous studies (Duvekot et al., 2018; Hallett et al., 2010; Pickard et al., 2017). In the context of ASD, the severity of social impairments has been linked to exposure to negative social experiences, such as bullying (Maïano et al., 2016; Schroeder et al., 2014), which is a well-known risk factor for the presence of mental health problems (Hoover & Kaufman, 2018; Stroud, 2018). Conversely, mental health difficulties also have an impact on social functioning. For example, psychopathology has been shown to be systematically related to the quality of everyday social interactions (Achterhof et al., 2022a). In the present study, we observed more specifically that individuals with externalizing problems had more social difficulties. This more specific link between externalizing problems and social impairments is not surprising and has already been highlighted in previous studies both in the general population and in ASD (Racz et al., 2017; Shea et al., 2018; Simonoff et al., 2008). Furthermore, it appears that it is the cumulative effects of psychiatric comorbidities, rather than one specific type of psychiatric comorbidity, that more significantly predicts difficulties in social functioning. Moreover, the rate of psychotropic medications was equivalent in both profiles, even though Profile 2 is associated with more psychiatric comorbidities. This highlights that mental health difficulties are probably underestimated in this Profile 2, a phenomenon frequently observed in individuals with ASD. Indeed, due to overlapping symptoms and ambiguous presentation of psychiatric conditions in ASD, assessment of co-occurring disorders is particularly complex (Mazefsky et al., 2012; Rosen et al., 2018). This diagnosis overshadowing can also have a negative impact on social functioning, as the resulting difficulties go unaddressed. It should also be noted that one of the parent-reported variables of social functioning – the ERSSQ – not only assessed social skills, but also emotional regulation (e.g., appropriate control of anxiety at home, at school or in the workplace). We can therefore imagine that this variable, and the differences observed between the two profiles on this variable, are also influenced by mental health. On the other hand, our results show that the self-evaluation (EMA) and direct observations by examiners (SSPA) are largely similar between the two profiles. This could indicate that mental health does not seem to have an impact on all aspects of social functioning. A second hypothesis could be that our two ASD profiles present social difficulties of similar intensity, but that their visibility varies according to the presence of mental health difficulties. Indeed, as the EMA and SSPA variables do not differ between the profiles, the absence of mental health difficulties would enable a better social adaptation in daily life, particularly as perceived by external observers (e.g. parents). Thus, individuals in Profile 1 could effectively mask their “true” social difficulties, given that they encounter fewer mental health difficulties. Conversely, individuals in Profile 2, with more mental health problems, would have more trouble masking difficulties in their social functioning. In short, this illustrates that masking and mental health difficulties can interact in complex ways, influencing and reinforcing each other (Cremone et al., 2023; Ross et al., 2023).

### Strengths, Limitations and Future Directions

The current study is the first to investigate social functioning profiles through a multi-method approach and in a homogeneous subgroup of autistic adolescents and young adults without intellectual disability. Some methodological limitations should however be considered. From a statistical point of view, and despite the differences observed between the profiles, as well as the relatively homogeneous sample and the size of the identified profiles that is in line with the recommendations of the goodness-of-fit model, the size of our sample was limited compared with other studies using latent profile analysis. Although there is no formal sample size recommendation in the literature (Spurk et al., 2020), the results should be replicated in larger, independent samples. Secondly, we draw the reader’s attention to the fact that the use of a wide range of measures based on standardized European and North American scales may have introduced variations in the results and influenced their interpretation. Indeed, as social functioning is highly context-dependent, it is also culture-dependent (Chen, 2012). Thirdly, since items from the EMA protocol are branched (i.e. depending on whether participants were alone or with others), some variables may be influenced by participants’ missing responses. For instance, a participant who never reported being in the company of others did not have the opportunity to assess the pleasantness of other people’s company or the level of interaction. In addition, the level of interaction was assessed with EMA through a single item (“We’re doing something together”). However, several additional indicators could be used to better assess this level of interaction, such as the content of joint activities and the fluidity of exchanges. In view of our results, it would be worth exploring this variable in greater details in future studies. Lastly, it is important to note that our sample consisted mainly of adolescents and young adults with a late diagnosis of ASD. Recent studies suggest that a delayed autism diagnosis, particularly in autistic women, could increase awareness of autistic traits and the need for masking (Begeer et al., 2013; Milner et al., 2023). This late diagnosis probably influenced the composition of our sample, in particular by increasing the proportion of women. The literature shows that women are often diagnosed later (Green et al., 2019), which could explain the close to 50:50 sex ratio in our study. In addition, receiving an ASD diagnosis later in life could have impacted our social functioning measures, particularly those that are parent-reported, as these individuals may be more likely to mask their social difficulties. Consequently, our results cannot be generalized to a population with an earlier diagnosis. It is essential that future studies focusing on social functioning take variables such as masking and age at diagnosis into account. At last, although individuals from the two profiles did not differ in terms of IQ, there could potentially differ in terms of specific cognitive processes. For example, the existence of these profiles could also be underpinned by fundamental attentional processes. Studies have shown an association between time spent exploring the face and eye regions of a face and better social functioning, as well as less severe autism symptoms (Riddiford et al., 2022). Additionally, variations in attentional processes toward social stimuli could account for both the observed distinctions between ASD profiles and the control group, as well as the divergences within the ASD profiles. For instance, individuals with ASD might consciously engage in eye contact and display emotions during interactions, even if they find eye contact uncomfortable and believe their emotions to be inauthentic (Hull et al., 2017; Lai et al., 2019). Consequently, more effective social attention in individuals from Profile 1 could also elucidate superior masking skills. Furthermore, differences in attentional processes could also be influenced by mental health difficulties due to their impact on emotional regulation processes (Joormann & D’Avanzato, 2010; Joormann & Vanderlind, 2014). This, in turn, would ultimately lead to compromised social functioning. Thus, *Masking* strategies, impacted by the presence of mental health difficulties, as well as attentional processes, often impacted in ASD, could influence the variations observed in social functioning and contribute to the distinctions observed between ASD profiles and the control group. Recognizing and understanding the complex link between social difficulties and emotional regulation difficulties therefore seems crucial in future studies to better understand their respective impact on mental health and social functioning.

## Conclusion

Considering the ubiquity of social interactions in daily life, and the negative long-term consequences that follow when social difficulties are present, this study aimed to improve the characterization of social functioning in a homogeneous group of autistic youths without intellectual disability. Given the inherent heterogeneity of ASD, it is essential to understand these variations in order to propose targeted and better-adapted interventions. For example, individuals with ASD who are more socially withdrawn might benefit from peer-mediated interventions aimed at encouraging them to engage in social interactions. Conversely, individuals with ASD who interact less during social situations could benefit from interventions focusing on initiating interaction, or on what to talk about during interaction. In both cases, to minimize the impact of mental health difficulties on social functioning, the deployment of adapted clinical care and training for emotional regulation strategies is indicated.

A multi-method approach enabled us to capture as many different aspects of social functioning as possible, and through different points of view. This led to the identification of two profiles within a homogeneous group of individuals with ASD. The social functioning of these two profiles is similar in some aspects to that of the control group, and different in others: both profiles show impaired social skills and a more negative evaluation of social interactions. In addition, compared with controls, differences in terms of involvement in social interactions were observed between the two profiles. However, the two profiles were only distinguished by their severity of social difficulties assessing by parents. It is worth noting that both profiles have similar IQ levels, suggesting that social difficulties are not related to cognitive abilities. Surprisingly, differences were also not attributed to gender, age, or socialization context as we could have expected. Moreover, parent-reported measures revealed qualitative distinctions, underscoring the importance of diverse assessment methods. Finally, mental health difficulties are common in people with ASD, particularly in those with more pronounced social difficulties, illustrating the bidirectional link between mental health and social functioning.
